# Noscapine targets EGFR^p-Tyr1068^ to suppress the proliferation and invasion of MG63 cells

**DOI:** 10.1038/srep37062

**Published:** 2016-11-10

**Authors:** Ming He, Linlin Jiang, Zhaozhou Ren, Guangbin Wang, Jiashi Wang

**Affiliations:** 1 Department of Orthopedic Surgery, Shengjing Hospital of China Medical University, Shenyang, Liaoning, People’s Republic of China; 2 Department of Electrotheropy, Shenyang Medical College Affiliated Central Hospital, Shenyang, Liaoning, People’s Republic of China

## Abstract

Osteosarcoma, the most common primary malignant bone tumor, usually arises in the metaphysis of long bones. Amplification and mutation of the epidermal growth factor receptor (EGFR) gene represent signature genetic abnormalities encountered in osteosarcoma. Noscapine is a benzylisoquinoline alkaloid derived from the opium poppy *Papaver somniferum*. Recently several studies have suggested its anti-cancer effect in melanoma, ovarian cancer, gliomas, breast cancer, lung cancer, and colon cancer. However, the underlying molecular mechanism for its anti-cancer effect still remains unclear. In this paper, we found the mechanism of noscapine effectively suppressed proliferation and invasion of MG63 cell line by inhibiting the phosphorylation of EGFR and its downstream pathway.

Osteosarcoma is the most common form of primary malignant bone tumor found in children^1^,^2^. Patients without clinical signs of systematic spread show 5-year survival rates of 60–80%^3^, whereas patients with metastasis at diagnosis exhibit 5-year survival rates of 20–30%^4^. Rosen proposed the current therapeutic sequence for high-grade osteosarcoma and it is now internationally accepted^5^,^6^. Since then, the prognosis of patients has more or less stagnated and no significant therapy improvements have been achieved^7^. In the last 10 years, better knowledge of oncogenic processes in osteosarcoma has led to the development of new therapeutic approaches based on single new drugs or administered in combination with conventional chemotherapy^8^. As such, vigorous efforts are ongoing to either improve current therapy or to identify and strike new molecular targets for osteosarcom therapy^9^. A number of genes and pathways have emerged as attractive therapeutic targets for osteosarcom, such as PI3K/mTOR, c-Src and VEGF^10^.

Noscapine (Fig. 1A) has been widely studied for its no analgesia, sedation and respiratory depression as an antitussive. Since its structure has certain similarities with colchicine, in its anti-tumor activity screening process found to have some anti-tumor activity through inhibiting tubulin. As a natural anti-cancer agent, noscapine was recently reported to exhibit activity against a variety of cancers through a poorly understood mechanism^11^.

The amplification of the epidermal growth factor receptor (EGFR) is one of the most common genetic aberration associated with malignant osteosarcoma^12^. EGFR is a cell membrane receptor with intrinsic protein tyrosine kinase activity that has been the subject of rigorous investigation in view of its involvement in several human cancers and its potential as a target of therapy^13^. Phase I clinical trials of EGFR tyrosine kinase inhibitors, such as gefitinib, have included patients with osteosarcoma^13^. EGFR has been shown to be important for cell proliferation and migration as well as in tumor progression^14^. The raise of EGFR^p-Tyr1068^ increased the levels of Cyclin D1 expression and promoted cell proliferation^15^. Inhibition of EGFR/Akt pathway could induce cell apoptosis. MMP2, as the downstream molecule of MEK-1/ERK1/2 pathway, is regulated by EGFR and plays important role in tumor invasion^16^.

Here we report that noscapine could effectively suppress the proliferation and invasion of MG63 cells, promote its early apoptosis by inhibiting the phosphorylation of EGFR.

## Materials and Methods

### Patients and tissue samples

15 matched osteosarcoma cancer and corresponding normal tissues were obtained from patients undergoing resection of tumor in the Shengjing Hospital of China Medical University. All patients had a clear histologic diagnosis of osteosarcoma cancer based on the AJCC. All cases were diagnosed with osteosarcoma cancer and treated between January 2010 and December 2010 at the Shengjing Hospital of China Medical University. The study protocol was approved by Medical Ethics and Human Clinical Trial Committee. All the tissues were immediately frozen in liquid nitrogen after surgery for research. We confirm that all methods were performed in accordance with the relevant guidelines and regulations. We confirm that informed consent was obtained from all subjects.

### Cell culture

Osteosarcoma cell lines MG63 and U2OS were obtained from American Type Culture Collection (Manassas, VA, USA). Cells were cultured in DMEM (Invitrogen, Carlsbad, CA, USA) containing 10% fetal calf serum (Invitrogen, Carlsbad, CA, USA). Cells were cultured at 37 °C with humidified atmosphere of 5% CO_2_.

### MTT assays

Cells were plated in 96-well plates (1 × 10^4^/well). 24 hours later, the medium was replaced with 100 ul medium containing different concentrations of noscapine and cultured for 0, 12, 24, 36 and 48 h. 20 μl of 5 mg/ml MTT solution was added to well. After incubation for 4 hours, the medium was removed and remaining MTT formazan was dissolved in 150 μl of DMSO. Solution was measured at 490 nm using a Microplate Reader (BIO-RAD).

### Cell-cycle analysis by flow cytometry

After incubation with different concentrations of noscapine, MG63 cells were washed using cold PBS and suspended in staining buffer with 10 ug/ml propidium iodide. The cells were analyzed using BD FACS Vantage flow cytometer (Becton Dickinson) and analyzed using CellQuest software program.

### Cell migration and invasion assay

Matrigel cell invasion assay was performed using a 24-well transwell chamber (8-μm pore size) (Corning). The transwell chambers were coated with 20 μl matrigel (1:4, BD Bioscience). Transfected cells were trypsinized and suspended in 100 μl of DMEM medium without serum, which was transferred to the upper chamber. 600 μl culture medium with 10% FBS was added to the lower chamber. After 18 hours incubation, the non-invaded cells on the upper membrane surface were removed using cotton swab and the cells that passed through the filter were fixed and stained with hematoxylin. The numbers of cells that pass through the membrane were counted in using microscope. This experiment was performed in triplicate.

### Cell apoptosis

Apoptosis was examined using Annexin-V-PI apoptosis detection kit. MG63 cells are washed with cold PBS and suspended using binding buffer. 5 μl of Annexin-V-FITC and 10 μl of PI were added. Cells were incubated in the dark for 15 minutes. 400 μl of binding buffer was added and the apoptosis rate was examined using BD FACS Vantage flow cytometer (Becton Dickinson).

### Reverse transcription and quantitative real-time PCR

Quantitative real-time PCR was performed using SYBR Green PCR master mix (TAKARA) in a total volume of 20 μl on Mx 3000 P Real-Time PCR System as follows: 95 °C for 30 seconds, 50 cycles of 95 °C for 10 seconds, 60 °C for 30 seconds. A dissociation step was performed to generate a melting curve to confirm the specificity of the amplification. β-actin was used as the reference gene. The relative levels of gene expression were calculated by the 2^−ΔΔCt^ method. Primer sequences were synthesized as the Table 1.

### Transfection of siRNA

Cells were transfected using pRS-siEGFR (Shanghai GeneChem Company) and then selected with puromycin (1.5 μg/mL) for 2 weeks. The cells transfected with pRS-si-NC (negative control) and cells without treatment were used as the control.

### Western blot analyses

Whole cell extracts were prepared using cell lysis buffer and about 60 μg of protein was separated using 10% SDS-polyacrylamide gels. After electrophoresis, the proteins were eletrotransferred to nitrocellulose filters and membranes was blocked using 5% nonfat dry milk in TBST for 2 hours. The proteins were probed with specific antibodies—CyclinD1, CDK4, CDK6, MMP2, Flag (Bioworld), EGFR, phospho-EGFR(Tyr1068), Akt, phospho-Akt(Ser473) (Santa Cruz), Caspase3, Bax and Bcl-2 (Neomarker). To assaure equal loading, gels were stripped and reprobed with antibodies against β-actin GAPDH (Kangchen Bio-tech Inc., Shanghai, China). All PVDF membranes were detected by chemiluminescence (ECL, Pierce Technology).

### Kinase Glo Luminescent assay

Indicated concentration of noscapine and 1 μL of HER1/2/3/4 kinases (Invitrogen) were mixed and filled with kinase buffer (50 mmol/L HEPES, pH 7.5, 10 mmol/L MgCl_2_, 2 mmol/L MnCl_2_ and 0.2 mmol/L DTT) to 20 μL. Incubated the mixture at 37 °C for 3 h then mixed with 20 μL Kinase Glo Luminescent (Promega) reaction solution, taking black 384-fluorescence values measured after standing 10 min.

### Immunoprecipitation and Kinase Glo Luminescent assay

MG63cells were transfected with Flag-EGFR-WT (wild type) or Flag-EGFR-KM (T1068A)(the kinase mutant of Tyr to Ala) after 24 h cells were washed with ice-cold PBS and suspended in cold lysis buffer supplemented with protease and phosphatase inhibitors as described. Supernatants were preadsorbed with anti-Flag M2 magnetic beads (Sigma-aldrich) for 3 h at 4 °C. Then the beads were mixed and filled with kinase buffer (50 mmol/L HEPES, pH 7.5, 10 mmol/l MgCl_2_, 2 mmol/lMnCl_2_ and 0.2 mmol/l DTT) to 20 μl. Incubated the mixture at 37 °C for 3 h then mixed with 20 μl Kinase Glo Luminescent (Promega) reaction solution, taking black 384-fluorescence values measured after standing 10 min.

### Tumor xenografts in nude mice

The procedures were approved by institutional animal research ethics committee with reference to the Chinese Community guidelines for the use of experimental animals (No. 201309). We confirm that all methods were performed in accordance with the relevant guidelines and regulations. Six-week-old male Balb/c nude mice were purchased from the Laboratory Animal center of Liaoning Province, China. All the animals were given free access to sterilized food and water. 1 × 10^7^ MG63 cell were suspended in 200 μl of PBS and subcutaneously injected into animal. The mice were assigned into control and treatment groups and anesthetized with injection of 75 mg/kg ketamine and 10 mg/kg xylazine^17^. The mice were treated with intravenous injection of noscapine (5 mg/kg) or DMSO once daily (days 0–16). Tumors became palpable 3 days after xenografting. The mice were monitored for 16 d after tumor inoculation. Body weight and Mortality were recorded during experimental period.

## Results

### Noscapine inhibits the growth and the invasion of MG63 and U2OS cells

**Figure 1:**
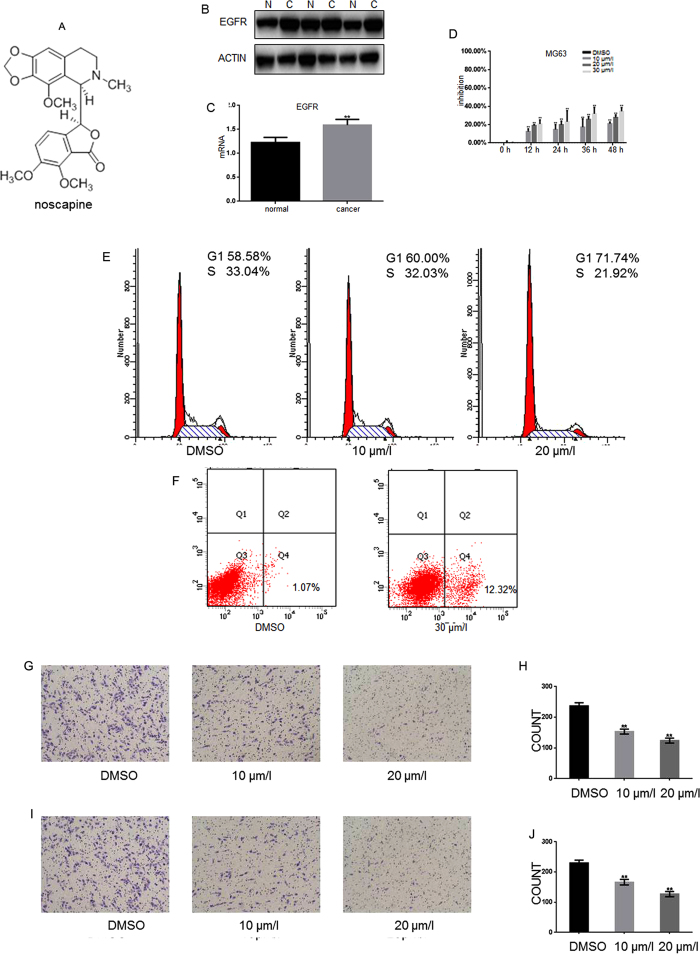
Noscapine inhibits the growth and the invasion of MG63 and U2OS (**A**) The structure of noscapine. (**B**) Protein level of EGFR in tissues was detected by western blot. (**C**) MRNA level of EGFR in tissues was detected by RT-PCR. *P < 0.05 vs. normal group, **P < 0.01 vs. normal group. (**D**) MG63cells were cultured with indicated concentrations of noscapine for indicated hours in 96-well plates, then MTT assay was performed, results represent the mean ± SD of three experiments done in triplicate. (**E**) MG63cells were pre-incubated with DMSO or noscapine for 24 h, then cells were analyzed using a FACS vantage flow cytometer with the Cell Quest acquisition and analysis software program (Becton Dickinson and Co., San Jose, CA), the experiment was repeated three times. (**F**) MG63cells were pre-incubated with noscapine for 24 h then cells were treated with ANNEXIN-V-FITC apoptosis detection kit and analyzed with FCAS. The experiment was repeated for three independent times. (**G,H**) MG63cells were pre-incubated with noscapine for 24 h; transwell assay without matrigel was performed. Cells were counted and results represent the mean ± SD of three experiments. *P < 0.05 vs. DMSO treated group, **P < 0.01 vs. DMSO treated group. (**I,J**) MG63cells were pre-incubated with noscapine for 24 h; transwell assay with matrigel was performed. Cells were counted and results represent the mean ± SD of three experiments. *P < 0.05 vs. DMSO treated group, **P < 0.01 vs. DMSO treated group.

Through the detection of protein and RNA levels, we found that the EGFR in osteosarcoma tissue was higher than that in adjacent tissues (Fig. 1B,C). The inhibitory effects on growth of noscapine were detected in MG63 and U2OS cells. As shown in MTT assay, noscapine inhibited the proliferation of MG63 and U2OS cells in a concentration dependent manner (Figs 1D and 6B). To further study the mechanisms of noscapine inhibiting the growth of osteosarcoma cells, MG63 cells were exposed to the indicated concentrations of noscapine for 24 h, and then cell cycle analysis was performed. Noscapine prominently induced a dose-dependent increase in the percentage of cells in G1 phase and a decrease in S phase compared with the control (Fig. 1E), indicating that noscapine arrest MG63 cells at the G1 phase of the cell cycle. To test whether noscapine could induce apoptosis of MG63cells, we detected the apoptosis rate by Annexin-V-FITC. By Annexin-V-FITC staining, the noscapine-induced MG63 cell apoptosis was increased with the increased concentration (Fig. 1F). Inhibitory effect of noscapine on migration and invasion of MG63cell was analyzed by transwell assay (with or without matrigel). Results showed that noscapine significantly decreased invasion and migration potential of osteosarcoma cells MG63 and U2OS(Figs 1G–J and 6E,F) in a dose-dependent manner. The above results showed that noscapine can inhibit the growth and the invasion of MG63 cells in a concentration dependent manner.

### Noscapine suppresses the kinase acivity of EGFR by inhibiting EGFR^p-Tyr1068^ in MG63 and U2OS cells

**Figure 2:**
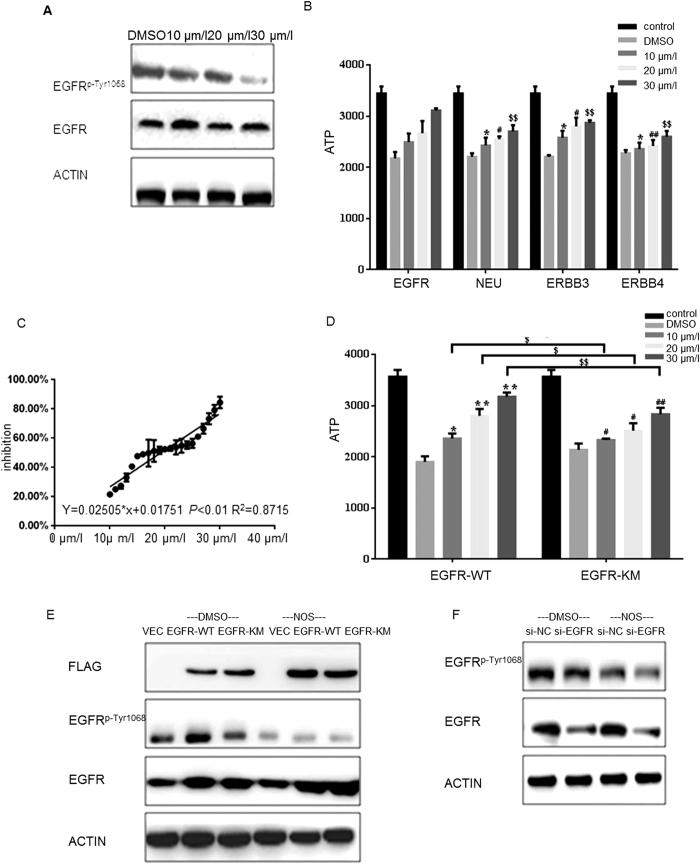
Noscapine inhibits the kinase acivity of EGFR by inhibiting EGFR^p-Tyr1068^ (**A**) MG63cells were treated with DMSO alone or indicated concentration of noscapine for 24 h, proteins were extracted and subjected to western blot analysis, the membrane was probed sequentially with EGFR and EGFR^p-Tyr1068^ antibody. (**B**) HER1/2/3/4 kinases were pre-incubated with the indicated concentrations of noscapine for 1 hour respectively, then Kinase Glo assay were performed. Data are shown as mean ± SD. *P < 0.05 vs. 10 μmol treated EGFR group; **P < 0.01 vs. 10 μmol treated EGFR group; ^#^P < 0.05 vs. 20 μmol treated EGFR group; ^##^P < 0.01 vs. 20 μmol treated EGFR group; ^$^P < 0.05 vs. 30 μmol treated EGFR group; ^$$^P < 0.01 vs. 30 μmol treated EGFR group.(**C**) EGFR kinase were pre-incubated with the indicated concentrations of noscapine for 1 hour respectively, then Kinase Glo assay were performed and inhibition rate were calculated, linear fit curve was drawn with an equation of y = 0.02505x + 0.01751 R^2^ = 0.8715. (**D**) Flag-EGFR-KM and Flag-EGFR-WT proteins were pre-incubated with the indicated concentrations of noscapine for 1 hour respectively, then Kinase Glo assay were performed. Data are shown as mean ± SD. *P < 0.05 vs. DMSO treated Flag-EGFR-WT group; **P < 0.01 vs. DMSO treated Flag-EGFR-WT group; ^#^P < 0.05, ^##^P < 0.01 vs. DMSO treated Flag-EGFR-KM group; ^$^P < 0.05, ^$$^P < 0.01 vs. Flag-EGFR-WT group treated Flag-EGFR-KM group; (**E**) MG63cells expressing Flag-vector, Flag-EGFR-KM or Flag-EGFR-WT and then treated with or without noscapine were detected with indicated antibody by western blot. (**F**) MG63cells transfected with si-EGFR or si-NC (negative control) and those cells were treated with or without noscapine were detected with indicated antibody by western blot.

Because of the important role of EGFR phosphorylation in the signal pathway, we wonder whether noscapine could inhibit the phosphorylation level of EGFR and suppress the activation of EGFR in MG63cells and U2OS cells. By western blot analysis, we found that treatment of noscapine could markedly inhibit the level of EGFR^p-Tyr1068^ but without affected the total expression of EGFR (Figs 2A and 6A). To further detect the selective inhibition profile of noscapine, we chose four types of kinase- EGFR/ErbB1/HER1, ErbB2/HER2/neu, ErbB3/HER3 and ErbB4/HER4; all of them are belong to the epidermal growth factor receptor family, which share a certain homology, to test the inhibition effect of noscapine on their kinase activation. By Kinase-Glo® Luminescent Kinase Assays (Promega) which is a homogeneous non-radioactive methodin vitro, we got the kinase inhibition rate of noscapine (Fig. 2B). The results showed that noscapine markedly inhibited EGFR kinase activity in a dose-dependent manner, but had relatively weak effect on the other three kinases, there were statistically significant difference between EGFR and the other three kinases at different concentration of noscapine (Fig. 2B). Then the inhibitory effect of noscapine on EGFR kinase was also tested by the assay. Data showed that noscapine inhibited EGFR activity with an IC_50_ value of 19.26 μmol/l (Fig. 2C).

To further verify the specific targeting of noscapine on EGFR, we performed two group experiments. Firstly, MG63 cells were transfected with Flag-vector, Flag-EGFR-WT (wild type) and Flag-EGFR-KM respectively and treated with the indicated noscapine for 24 h, the kinase inhibition rate of noscapine between EGFR-WT and EGFR-KM were compared by Kinase-Glo® Luminescent Kinase Assays. The results showed that noscapine markedly inhibited EGFR-WT kinase activity in a dose-dependent manner, but had relatively weak effect on EGFR-KM (Fig. 2D). Then the indicated proteins level was tested by western blot analysis (Fig. 2E). As the results showed, in the DMSO treated control group, the level of EGFR^p-Tyr1068^ in cells expressed Flag-EGFR-KM was significantly less than that in the cells expressed Flag-EGFR-WT. In the noscapine treated group, in both cells expressed Flag-EGFR-WT and Flag-EGFR-KM, the level of EGFR^p-Tyr1068^ dramatically decreased compared with the DMSO treated control group, but the total EGFR level were not changed(Fig. 2E).

In another set of experiments, the MG63/si-NC (negtive control) stable cell line and MG63/si-EGFR stable cell line were treated with or without noscapine, the level of EGFR^p-Tyr1068^ and total EGFR were tested by western blot analysis (Fig. 2F). The data showed that the phosphorylation degree of EGFR^p-Tyr1068^ significantly reduced after noscapine treatment in MG63/si-NC stable cell lines but not changed significantly in MG63/si-EGFR stable cell line compared with the DMSO control group.

The above results indicated that noscapine suppressed the kinase acivity of EGFR, at least partly by inhibiting the phosphorylation of EGFR^p-Tyr1068^ in MG63 cells.

### Noscapine inhibits the growth of MG63 and U2OS by inhibiting EGFR/Akt/CDKs and EGFR/Akt/Bad pathway

**Figure 3:**
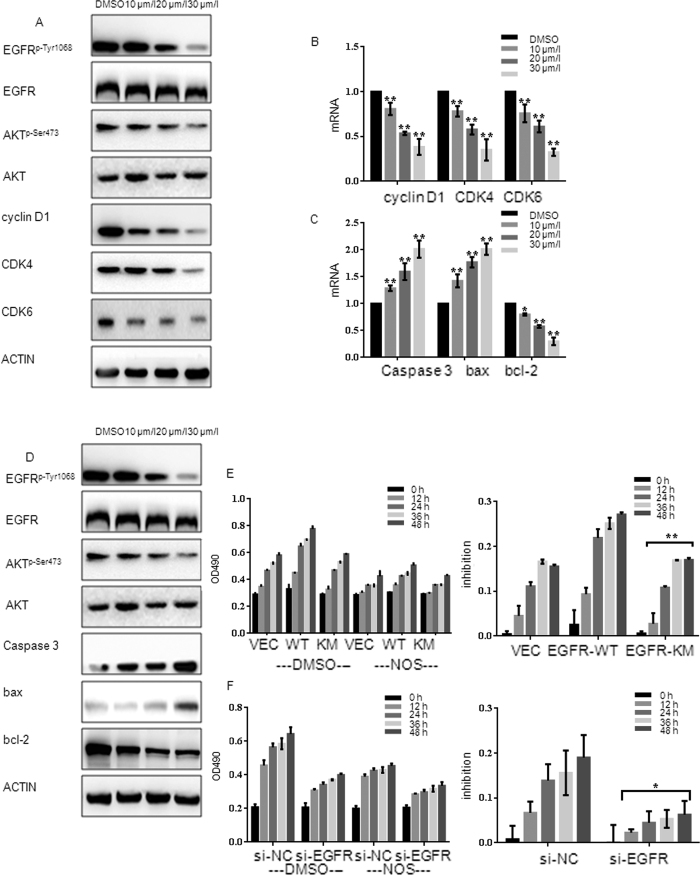
Noscapine represses the growth of MG63cells by inhibiting EGFR/Akt/CDKs and EGFR/Akt/Bad pathways (**A**) MG63cells were treated with DMSO alone or indicated concentration of noscapine for 24 h, the protein expression of Cyclin D1, CDK4, CDK6, EGFR, EGFR^p-Tyr1068^, Akt, and Akt^p-Ser473^ were detected by western blot. (**B**) MG63cells were treated with DMSO alone or noscapine for 24 h, then cells were harvested, and the mRNA expression of Cyclin D1, CDK4 and CDK6 were detected by real-time RT-PCR; results represent the mean ± SD of three experiments done in triplicate. *P < 0.05 vs. DMSO treated group, **P < 0.01 vs. DMSO treated group. (**C**) MG63cells were treated with DMSO alone or indicated concentration of noscapine for 24 h, cells were harvested, and the mRNA expression of Caspase3, Bax and Bcl-2 were detected by real-time RT-PCR, results represent the mean ± SD of three experiments done triplicate. *P < 0.05 vs. DMSO treated group, **P < 0.01 vs. DMSO treated group. (**D**) MG63cells were treated with DMSO alone or indicated concentration of noscapine for 24 h, the protein expression of Caspase3, Bax, Bcl-2, EGFR, EGFR^p-Tyr1068^ Akt, and Akt^p-Ser473^ were detected by western blot. (**E**) MG63 cells expressing Flag-VECTOR, Flag-EGFR-KM or Flag-EGFR-WT were incubated with 20 μmol/l of noscapine, MTT assay was performed after indicated hours and results represent the mean ± SD of three experiments done in triplicate. **P < 0.01 vs. Flag-Akt-KM/Flag-Akt-WT. (**F**) MG63 cells transfected with si-EGFR or si-NC (negative control) were treated with 20 μmol/l of noscapine, MTT assay was performed after indicated hours and results represent the mean ± SD of three experiments done in triplicate. *P < 0.05 vs. si-EGFR/si-NC (negative control). (**G**) MG63cells expressing Flag-vector, Flag-EGFR-KM or Flag-EGFR-WT and then treated with or without noscapine were detected with Cyclin D1, CDK4, CDK6, EGFR, EGFR^p-Tyr1068^, Akt and Akt^p-Ser473^ antibody by western blot. (**H**) MG63cells expressing Flag-vector, Flag-EGFR-KM or Flag-EGFR-WT and then treated with or without noscapine were detected with Caspase3, Bax, Bcl-2, EGFR, EGFR^p-Tyr1068^, Akt and Akt^p-Ser473^ antibody by western blot.

Since Cyclin D1, CDK4 and CDK6 are key regulators in the G_1_ phase of the cell cycle, here we examined the indicated regulators expression level in noscapine-treated cells. Western blot analysis showed that exposure of MG63 and U2OS to 10/20/30 μmol/l noscapine for 24 h dramatically decreased the expression of Akt, Akt^p-Ser473^, Cyclin D1, CDK4 and CDK6 (Figs 3A and 6C), indicating noscapine arrests cells at G1 phase and then suppresses cells growth via down-regulated Akt^p-Ser473^, Cyclin D1, CDK4 and CDK6. Furthermore, real time RT-PCR showed that expression of Cyclin D1, CDK4 and CDK6 in MG63 were down-regulated at mRNA level after exposure to noscapine (Fig. 3B). Furthermore the expressions of apoptosis regulators were also examined by western blot. The expression of Bcl-2 was obviously decreased and the levels of Caspase3 and Bax were increased in noscapine treated MG63 and U2OS cells (Figs 3D and 6D). Then real time RT-PCR results verified that changes of these factors were coincidence with protein levels (Fig. 3C).

To further confirm the involvement of EGFR in noscapine -induced MG63 cells growth arrest, cells respectively expressed Flag-EGFR-WT and Flag-EGFR-KM plasmids were treated with 20 μmol/l noscapine for 24 h. Then cell proliferation was analyzed by MTT assay. Results showed that the proliferation of cells expressed EGFR-WT was dramatically inhibited by noscapine compared with cells transfected with EGFR-KM plasmid, but there was no significant difference in the inhibition rate between EGFR-KM expressed cells and cells transfected with vector cells (Fig. 3E). Then, the same experiments were performed in MG63/si-NC (negtive control) cell line and MG63/si-EGFR cell line. Results showed that, there was significantly difference in the proliferation inhibition rate between MG63/si-NC and MG63/si-EGFR cell lines (Fig. 3F). While, western blot analysis showed that under the effect of noscapine, when the Akt^p-Ser473^ was over-expression EGFR^p-Tyr1068^, Cyclin D1, CDK4, CDK6, Bcl-2, Caspase3 and Bax changed more significantly (Fig. 3G,H).

### Noscapine represses the migratory and invasive potential of MG63 by inhibiting EGFR/Akt/MMP2 pathway

**Figure 4:**
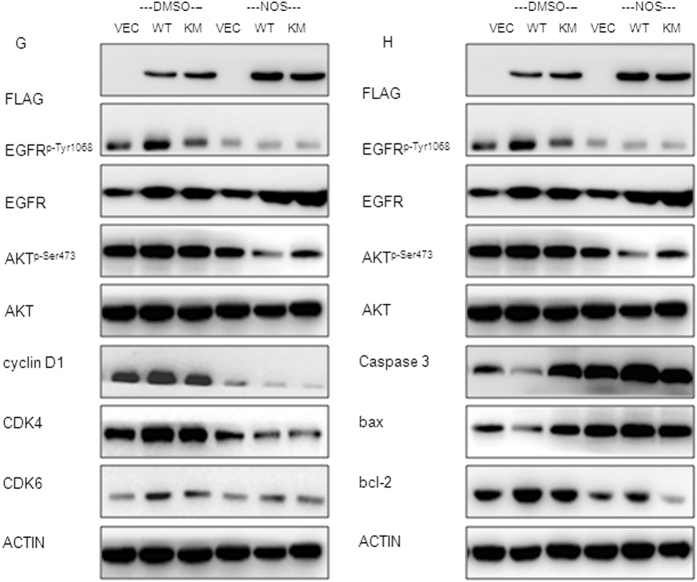
Noscapine represses the migratory and invasive potential of MG63cells by inhibiting EGFR/Akt/MMP2 pathway (**A**) MG63cells were treated with DMSO alone or indicated concentration of noscapine for 24 h, the protein expression of EGFR, EGFR^p-Tyr1068^, Akt, Akt^p-Ser473^ and MMP2 were detected by western blot. (**B**) MG63cells were treated with DMSO alone or indicated concentrations of noscapine for 24 h, cells were harvested, and the mRNA expression of MMP2 was detected by real-time RT-PCR, results represent the mean ± SD of three experiments done in triplicate. *P < 0.05 vs. DMSO treated group; **P < 0.01 vs. DMSO treated group. (**C,D**) MG63 cells expressing Flag-vector, Flag-EGFR-KM or Flag-EGFR-WT and then treated with or without noscapine were detected by transwell without matrigel assay. The experiment was repeated for three independent times. (**E**) MG63cells expressing Flag-vector, Flag-EGFR-KM or Flag-EGFR-WT and then treated with or without noscapine were detected with EGFR, EGFR^p-Tyr1068^, Akt, Akt^p-Ser473^ and MMP2 antibody by western blot.

To further investigate the mechanisms of noscapine migratory of osteosarcoma cells, MG63 and U2OS cells were exposed to various concentrations of noscapine for 24 h. Western blot analysis showed that levels of MMP2 dramatically decreased (Figs 4A and 6G). Real time RT-PCR showed that expression of MMP2 in MG63 was down-regulated at mRNA level after exposure to noscapine (Fig. 4B). The migration capacity was detected in EGFR-WT, EGFR-KM (kinase mutation EGFR) and MG63/si-EGFR cell lines with or without noscapine. And results suggested that EGFR is the target of noscapine (Fig. 4C,D). Western blot analysis showed that under the effect of noscapine, when the EGFR was over-expression EGFR^p-Tyr1068^ and MMP2 decreased more significantly (Fig. 4E).

### The anti-tumorigenic effect of noscapine *in vivo*

**Figure 5:**
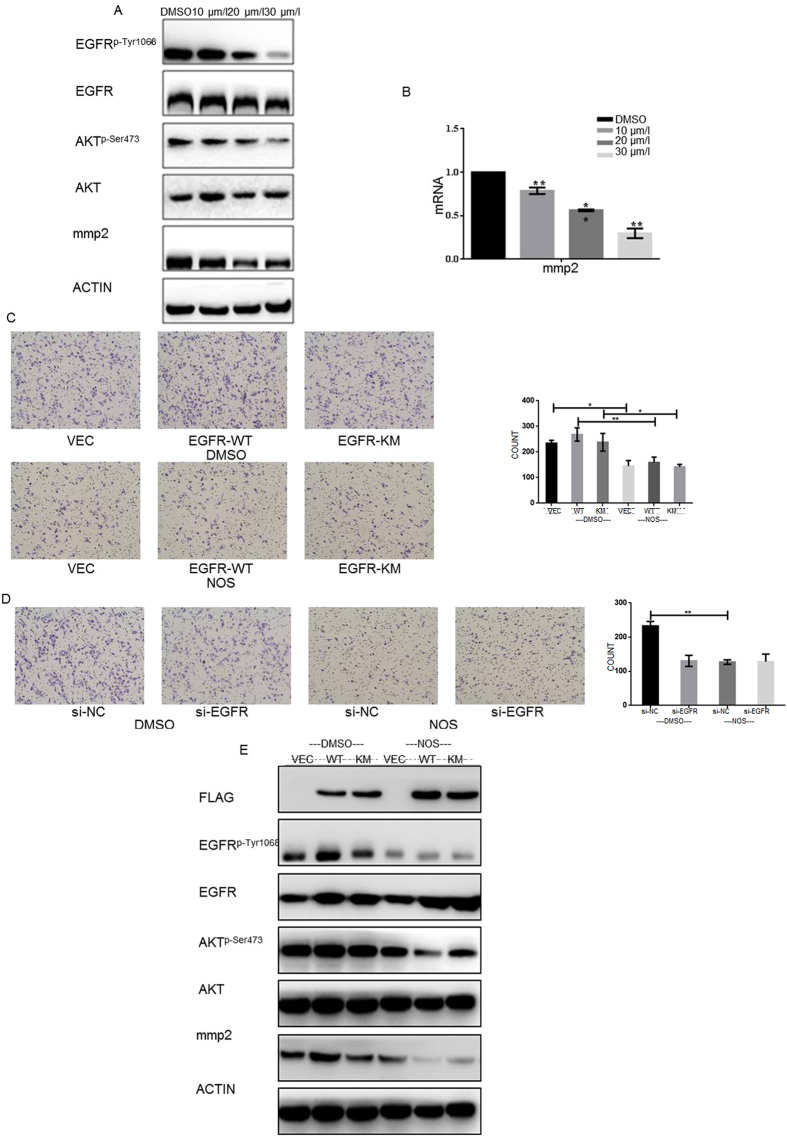
(**A**) Photographic illustration of tumors from control and noscapine -treated nude mice on the day of sacrifice (day 16). (**B**) The tumor weights of them were showed in the graph. (**C**) The protein expression of Cyclin D1, CDK4, CDK6, Caspase 3, Bax, Bcl-2, MMP2, EGFR, EGFR^p-Tyr1068^, Akt, and Akt^p-Ser473^ were detected by western blot. (**D**) The ideograph showed that noscapine effectively suppressed proliferation and invasion of MG63 cells by inhibiting EGFR, then inhibiting EGFR pathway.

**Figure 6:**
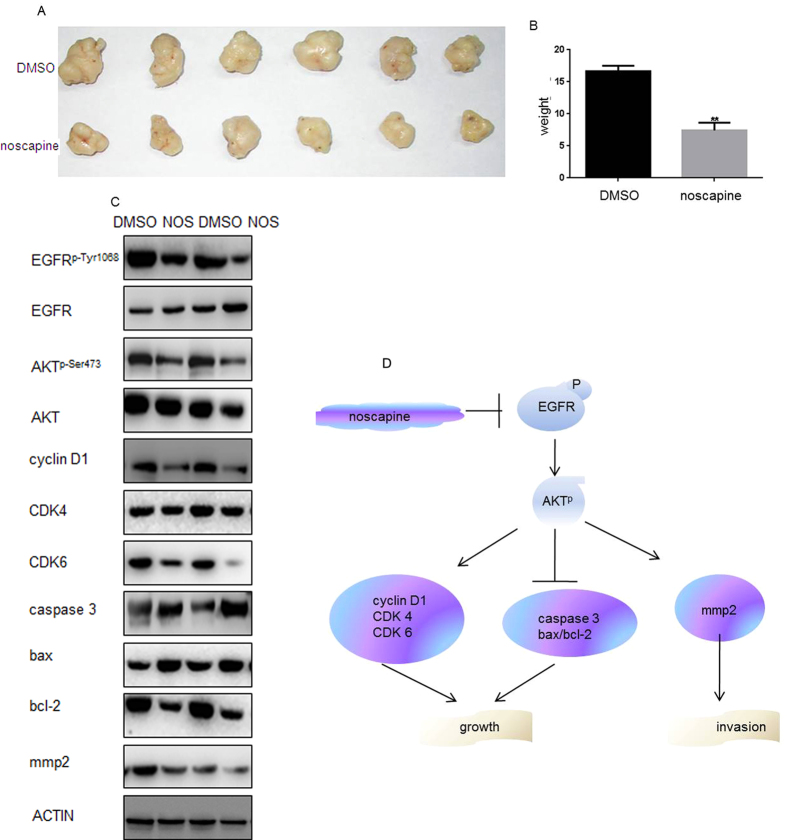
(**A**) The protein expression of EGFR, EGFR^p-Tyr1068^, Akt, and Akt^p-Ser473^ in U2OS cells were detected by western blot. (**B**) MG63cells were cultured with indicated concentrations of noscapine for indicated hours in 96-well plates, then MTT assay was performed, results represent the mean ± SD of three experiments done in triplicate. (**C,D**) The protein expression of Cyclin D1, CDK4, CDK6, Caspase 3, Bax and Bcl-2 in U2OS cells were detected by western blot. (**E**) U2OS cells were pre-incubated with noscapine for 24 h; transwell assay without matrigel was performed. Cells were counted and results represent the mean ± SD of three experiments. *P < 0.05 vs. DMSO treated group, **P < 0.01 vs. DMSO treated group. (**F**) U2OS cells were pre-incubated with noscapine for 24 h; transwell assay with matrigel was performed. Cells were counted and results represent the mean ± SD of three experiments. *P < 0.05 vs. DMSO treated group, **P < 0.01 vs. DMSO treated group. (**G**) The protein expression of MMP2 in U2OS cells were detected by western blot.

The anti-tumorigenic effect of noscapine on MG63 cells was further illustrated *in vivo* in a nude mouse xenogfraft. On the day of sacrifice (day 16), fangchinoline treatments at the given doses resulted in about 41.23% tumor suppression (Fig. 5A,B). Then the protein levels of EGFR and its pathways were detected by western blot (Fig. 5C). The above results suggested that noscapine inhibits the migratory and invasive of MG63 cells by inhibiting EGFR pathway. These results indicated that noscapine effectively suppressed proliferation and invasion of MG63 cells by inhibiting EGFR^p-Tyr1068^ (Fig. 5D).

## Discussion

In cancer cells, EGFR aberrations impact a variety of cell signaling pathways, notably the PI3K-AKT and JAK/STAT pathways^18^. In osteosarcoma, *in vitro* data from early passage osteosarcoma cells demonstrate constitutive EGFR phosphorylation whose abrogation leads to growth inhibition^19^. Overexpression of EGFR has been shown to promote cancer cell motility and invasion. *In vitro* data indicate that EGFR and Akt signaling play a role in the pathogenesis of osteosarcoma^20^.

Noscapine was shown to have potent antitumor activity against murine lymphoid tumors^21^. Since then, noscapine has been shown to exhibit activity against a wide variety of tumors *in vitro* and *in vivo*^22^,^23^,^24^,^25^. There are findings suggest that noscapine can promote apoptosis by suppressing Bcl-2^26^. Besides antiapoptotic proteins, noscapine also downregulates the expression of proteins linked to cell proliferation, inflammation, invasion, adhesion, and angiogenesis. These observations imply that noscapine has anti-inflammatory, antiangiogenic, and antimetastatic activities^27^. At the same time in hypoxic human glioma cells, noscapine has been shown to inhibit the secretion of VEGF^28^,^29^.

In this study, MG63cells were used to detect the anti-cancer effect of noscapine. As shown in MTT assay and transwell assay, noscapine treatment inhibited the proliferation and migration of MG63cells in a concentration-dependent manner. We found that the phosphorylation of EGFR (Tyr1068) dramatically decreased with the increasing concentration of noscapine, which suggested noscapine suppressed the phosphorylation of EGFR and inhibited the proliferation and migration of MG63cells. We first found that noscapine did suppress the phosphprylation levels of EGFR, so it is reasonable to conclude that noscapine suppressed Cyclin D1 and CDK4/6 expression via suppression of EGFR pathway, and inhibited the transition of cells from G1 phase to S phase, and resulted in the anti-proliferative effect on MG63 cells together with the induction of apoptosis.

In addition to the effect on cell proliferation, we demonstrated the inhibition mechanism of noscapine on invasion of MG63 cells. One of the key steps in cancer invasion and metastasis is the degradation of extracellular matrix. MMP2 has been demonstrated to play important roles in the process^30^. Our results showed that noscapine significantly suppressed the invasive ability of MG63cells in parallel with down-regulation of MMP2 and inhibit EGFR pathway.

In summary, our data showed that noscapine could inhibit the malignant phenotype of MG63 cells by inhibit the phosphorylation of EGFR (Tyr1068) and further to suppress the EGFR associated signaling pathway, EGFR/Akt pathway. Also the anti-tumorigenic effect of noscapine on MG63 cells was illustrated *in vivo*. Although these results are warranted further testing, the present findings do support the conception that noscapine may offer a novel therapeutic strategy for advanced metastatic osteosarcoma.

## Additional Information

**How to cite this article**: He, M. *et al*. Noscapine targets EGFR^p-Tyr1068^ to suppress the proliferation and invasion of MG63 cells. *Sci. Rep.* **6**, 37062; doi: 10.1038/srep37062 (2016).

**Publisher’s note:** Springer Nature remains neutral with regard to jurisdictional claims in published maps and institutional affiliations.

## Figures and Tables

**Table 1:** The primers of real-time PCR.

Name	Forward primer(5′->3′)	Reverse primer(5′->3′)
Cyclin D1	CCGAGGAGCTGCTGCAAATGGAGCT	TGAAATCGTGCGGGGTCATTGCGGC
CDK4	CAGAGCTCTTAGCCGAGCGT	GGCACCGACACCAATTTCAG
CDK6	AGTCTGATTACCTGCTCCGC	CCTCGAAGCGAAGTCCTCAA
Caspase3	TGTGAGGCGGTTGTAGAAGTT	GCTGCATCGACATCTGTACC
Bcl-2	GGTGAACTGGGGGAGGATTG	GGCAGGCATGTTGACTTCAC
Bax	AGCTGAGCGAGTGTCTCAAG	GTCCAATGTCCAGCCCATGA
MMP2	CGCATCTGGGGCTTTAAACAT	TCAGCACAAACAGGTTGCAG
β-actin	TCGTGCGTGACATTAAGGAG	ATGCCAGGGTACATGGTGGT
